# Drs. Peck and Allen

**Published:** 1885-12

**Authors:** Ad. Lippe

**Affiliations:** Philadelphia


### DRS. PECK AND ALLEN.

AD. LIPPE, M. D., PHILADELPHIA.

An extremely interesting paper is before us on pages 194 to 199 of the October number of the *Medical Advance.* George B. Peck, M.D., relates the impressions which the address of the President of the American Institute of Homœopathy made on him on the memorable second day of June, A. D. 1885 ; locality, the ordinary parlor of the Lindell Hotel, St. Louis; time, ten A. M.; President of the Institute and orator, Professor T. F. Allen, from New York. Dr. Peck describes his astonishment over that address, and gives Professor Allen's (?) answer to a letter by him addressed to the orator, in which answer Professor Allen attempts to set himself right before the profession, when he proposes to put Homœopathy to a new test, and the learned Professor is especially anxious to open again the posological question.

After mature reflection we come to the conclusion that Professor Allen has not been prudent in reopening this vexed question and in defining his own personal conclusions he arrives at after his own experiments. It is very evident that Professor Allen in his own statements gives us the reasons of the conclusions he arrives at. Formerly, says Professor Allen, he administered the two hundredth potency in water and could repeat the dose every hour or two with impunity. Now he claims that he has prescribed for the last few years the third and sixth, and says, " I get my best results from single doses, much better, indeed, than formerly from single doses of high potencies." Here is a palpable contradiction and a fatal error. The repetition of the two hundredth or of any potency indiscriminately or habitually cannot be made every hour or two with impunity, failures must follow necessarily, and if Professor Allen has better results from the third or sixth in *single* doses he commits a fatal error in condemning the higher potencies, as he administered them, by his own confession, indiscriminately and habitually every hour or two. Of course, he will have better results if he returns to the single dose, be it the third or sixth or two hundredth, or a much higher potency. But may we ask if Hahnemann and his methods are to be put on trial again, and that before a packed jury and incompetent judges? Has the posological question not been settled long ago? Have not the Vienna provers given a final verdict in favor of the non-sick-making and more curative properties of Natrum mur. in the thirtieth potency than in the lower potencies? In this case the judge and jury were, as Dr. Watzke confesses, prejudiced in favor of the lower potencies, but the facts were so strong, were obtained by honest prejudiced men, that, notwithstanding their prejudices, honest Dr. Watzke gave vent to his own sentiments in the *Œsterreiche Zeitschrift*, Vol. IV, page 251, where he says: "Concerning the nosology of our remedy (Natrum muriaticum), I am unfortunately—I say *unfortunately*—COMPELLED to declare myself in favor of the higher potencies; I would have preferred to represent the generally prevailing notion of the usually applied lower doses. The physiological provings, as well as supervening clinical results made with kitchen salt which have been obtained so far, speak decidedly and positively for them." The re-provings of Nat. mur. were made by skeptics, the provings were made honestly, and Hahnemann with his own *Materia Medica* was on trial before these skeptics; they were, much against their will and wish, *compelled* to indorse not only the great, wise man, Hahnemann, but both the physiological provings, and the clinical applications of the thirtieth potency overwhelmingly proved its superiority over the lower potency. This confession by skeptical Dr. Watzke was made in 1848, and in 1885 all the noble work of these honest skeptics and their confessions seem to be forgotten—or ignored. The President of the American Institute is again stirring up this burning question of posology, and in his address deliberately resorts to misstatements. The chasm widening year by year, as some professing homœopaths in Boston express it, is not a difference on the posological question, as President T. F. Allen impliedly asserts when he says: "The belief in them (the high potencies) has led to the formation of a society by some of our members who wish the most perfect freedom in expressing their opinions and relating their experiences." (*Transactions*, page 26.) President Allen here alludes to the formation of the International Hahnemannian Association. We take the liberty to contradict him flatly. The members of the I. H. A. were disgusted with the rapidly progressing departures in our homœopathic ranks tolerated and fostered by the American Institute; their original object was *to organize* in opposition to the abominable heresies tolerated by the Institute. There was not a syllable said about the imaginary division in our ranks—as high and low potency men. The members of the I. H. A. united themselves to uphold the Law of the Similars, the single remedy, and the minimum dose. Who but an idiot can interpret the minimum dose into "high potency"? It always stood for the dose "just sufficient to cure." The vexatious subject on which the younger men with no convictions are coming to us, is, that they should be held to comply with the Law of Cure, the methods of Hahnemann, etc. As long as the American Institute of Homœopathy sustains or even tolerates such utterly misleading and unhomœopathic books as that just published by a member of the Institute, Dr. Arndt—his *System of Medicine, etc.*, these young men will gain a conviction that Homœopathy and its practice are based on "no Law," and what will be the result? Why, they will turn out to be despised eclectics, governed by "no Law;" they will still more swell the ranks of pretending homœopaths, a disgrace to the medical profession, and if a president of the Institute tells them that practically there is a limit to the divisibility of drug power associated with material substances, and that this divisibility is very finite, they must be supposed to be utterly ignorant of what is going on in the scientific world. Shall Professor Jaeger and a host of scientists be set aside? Their discoveries must be ignored because they do not suit the President of the Institute, but Arndt's eclectic and unscientific work must be tolerated. Finally, the proverbial woolly headed Ethiopian emerges from the wood-pile. On page 28 of the *Transactions* of 1885 Professor Allen says: "It seems to many of us impossible to ignore the results of the high dilutions on the sick, but these apparent results must be confirmed by experiment on the healthy, and our practice made to conform to our positive knowledge."

These apparent (open, visible, evident) results must be confirmed by experiment on the healthy; exactly so; and till then these results must be set down as in appearance only—*illusive*, so says Professor Allen. Does the learned Professor expect to have all and every symptom or group of symptoms cured, permanently cured, by high potencies reproduced by proving the same so-called high potencies on the healthy? And will he persist in his refusal to accept the testimony of qualified witnesses till these proposed provings are made? The learned Professor has voluntarily given testimony to which nobody objects, as it has been given in good faith and to the best of the witness' knowledge and belief. We take the liberty to ascend to the witness stand, and hope such men as have the same experience will from time to time mount the witness stand and testify as well as they can to the best of their knowledge and belief. And we now state that the proposed experiments have been made not only in Vienna but just over the North River in the city of Brooklyn—to be explicit, there resides in the said city of Brooklyn a Dr. B. Fincke, who has made and published a proving made on the healthy with a very high attenuation (fluction potency) of Lachesis; and that I, a resident of Philadelphia, have noted down said provings; and that under the administration of single doses such symptoms as had been produced by Lachesis 5M on the healthy were cured on the sick, not once but repeatedly; that we have found Lachesis 5M curative in nymphomania if otherwise indicated; that we always held the clinical experiment to be the only true test of the reliability of the provings of a drug; that for forty years the experiment, with first the two hundredth, and later with much higher potencies, has resulted in increasingly favorable results provided the methods of Hahnemann were strictly followed; that long, painstaking experiments have shown that by means of potentiation the curative virtues of drugs are developed and increased; that a persistent denial of the correctness of such statements does not creditably reflect on a person making such denials. We have pretending homœopaths, and we know of public teachers, in so-called—erroneously so-called—Hahnemannian medical colleges, who unblushingly teach that intermittent fever does not admit of strictly homœopathic treatment, but that fifteen-grain doses of the crude Chininum sulph. is the proper dose to cure that disease. The fact remains the same that every case of intermittent fever can and must be cured if the truly homœopathic remedy is properly administered, and if Professor Allen claims that he obtains better results from the third and sixth potency than he did from the two hundredth, the reflecting physician must come to the conclusion that both the Quinine teacher and Professor Allen have stepped forward and unwittingly made a profession of a *testimonium paupertatis.* If, as Professor Allen says, on page 197 of the October number of the *Medical Advance*, "No amount of clinical evidence will ever be able to demonstrate these propositions (theories of dynamization and the action of infinitesimals) as truths of nature," the simple question arises, How does *he* demonstrate *his* proposition? Why, by his own individual clinical evidence, unsupported, to be sure, by anything but his own assertions; and by what authority does he undertake to set aside the ever-accumulating clinical evidence that dynamization and the action of the infinitesimals *are* truths of nature? The clinical experiment is the ONLY final test of the truths of nature, and if Professor Allen looks down upon this only possible test and the acceptance of it and the results obtained by it as *puerile* HE is welcome to that *opinion*. Finally, Professor Allen and everybody else must come down to the clinical test. How else can they ascertain the correctness of any proving of any drug in substance in lower, and finally in a highly dynamized form but by the clinical test? A very valuable beginning could be inaugurated by publishing a *correct* translation of the exhaustive provings of Natrum mur. by the Vienna provers to be found in the fourth volume of the *Œsterreiche Zeitschrift*, as also the deductions honest Dr. Watzke draws from said provings and from the clinical experiments. It may be as well to remember how the Milwaukee test fizzled out—how Gotham tried to revive isopathy, and how we hear no more of it, and as Gotham now proposes to put Hahnemann and his approved methods on trial again, it may be expected that this trial will end in a fizzle, while the proponent may well remember that a new trial cannot possibly be inaugurated till a previously obtained verdict is set aside. Dr. Watzke published that verdict, and it will require some very powerful argument to set it aside; it is for this reason that a publication of that previously obtained verdict is published, as also the reasons why it should be set aside and a new trial be called into existence by Professor T. F. Allen from Gotham.

